# Factors associated with the decision to undergo risk-reducing salpingo-oophorectomy among women at high risk for hereditary breast and ovarian cancer: a systematic review

**DOI:** 10.4069/kjwhn.2020.11.19

**Published:** 2020-12-09

**Authors:** Sun-young Park, Youlim Kim, Sue Kim

**Affiliations:** 1 Graduate School, Yonsei University, Seoul, Korea; 2 National Evidence-based Healthcare Collaborating Agency, Seoul, Korea; 3 Mo-Im Kim Nursing Research Institute and College of Nursing, Yonsei University, Seoul, Korea

**Keywords:** Decision making, Hereditary breast and ovarian cancer syndrome, Salpingo-oophorectomy, Systematic review

## Abstract

**Purpose:**

This systematic review aims to identify factors associated with risk-reducing salpingo-oophorectomy (RRSO), including the uptake rate and decision timing, among women at high risk for hereditary breast and ovarian cancer (HBOC).

**Methods:**

We found 4,935 relevant studies using MEDLINE, Embase, CINAHL, and PsycINFO on July 6, 2020. Two authors screened the articles and extracted data. Twenty-four studies met the inclusion criteria. Quality assessment of articles was conducted using the Risk of Bias for Nonrandomized Studies tool.

**Results:**

Five types of factors were identified (demographic factors, clinical factors, family history of cancer, psychological factors, and objective cancer risk). The specific significant factors were older age, having child(ren), being a *BRCA1/2* carrier, mastectomy history, perceived risk for ovarian cancer, and perceived advantages of RRSO, whereas objective cancer risk was not significant. The uptake rate of RRSO was 23.4% to 87.2% (mean, 45.2%) among high-risk women for HBOC. The mean time to decide whether to undergo RRSO after *BRCA* testing was 4 to 34 months.

**Conclusion:**

RRSO decisions are affected by demographic, clinical, and psychological factors, rather than objective cancer risk. Nonetheless, women seeking RRSO should be offered information about objective cancer risk. Even though decision-making for RRSO is a complex and multifaceted process, the psychosocial factors that may influence decisions have not been comprehensively examined, including family attitudes toward RRSO, cultural norms, social values, and health care providers’ attitudes.

## Introduction

Hereditary breast and ovarian cancer (HBOC) is a syndrome that is associated with an increased incidence of breast and ovarian cancers [1]. Approximately 12% of breast cancer and 1% to 2% of ovarian cancer cases occur in women with HBOC [2]. The most common causes of HBOC are mutations in the breast cancer susceptibility 1 and 2 genes (*BRCA1* and *BRCA2*, respectively) [1,2]. *BRCA* mutations are found in 15.7% of women with a personal or family history of breast/ovarian cancer in Korea [3]. Among *BRCA1* carriers, the average lifetime cancer risks are 67% for breast cancer and 45% for ovarian cancer. Among *BRCA2* carriers, these risks are 66% and 12% for breast and ovarian cancer, respectively. Therefore, clinical prevention options, such as risk-reducing salpingo-oophorectomy (RRSO), risk-reducing mastectomy (RRM), intensive surveillance for early detection of ovarian/breast cancer, and chemoprevention with tamoxifen, are offered to women at high risk for HBOC [1,2].

Among these preventive strategies, the most frequently offered option is RRSO because it reduces the risk of cancer incidence by more than 95% for ovarian cancer and 50% for breast cancer [4]. The National Comprehensive Cancer Network recommends that RRSO should be considered for women with *BRCA* mutations aged 35 to 40 years who have completed childbirth [5]. However, deciding to undergo RRSO is not an easy process and is affected by multifaceted factors [1,5,6]. Because RRSO not only causes postoperative menopause [1,5], but also negatively affects quality of life and psychological health, and can also alter one’s identity as a woman [5,7]. Previous studies have shown that women tend to overestimate their own cancer risk [8], to perceive information about cancer risk and preventive surgery as lacking [9], and to feel that there is pressure from clinical specialists to choose certain preventive options [10]. Therefore, genetic counseling should be improved to provide helpful decision-making support, and to do so, it is necessary to understand the current process of making decisions about RRSO [5,7].

After disclosure of the results of the *BRCA* test, it takes a long time for some women to select a preventive option [7], and RRSO rates have been reported to range from 13% in the USA to 75% in the Netherlands [11]. Therefore, a comprehensive exploration of the rate and timing of decision-making regarding RRSO is needed.

Although previous studies have shown that RRSO decision-making is influenced by demographic, clinical, psychological, and social factors [7,11-13], only one systematic review has integrated and explored various factors, and it did not identify significant factors [7]. Furthermore, perceptions of HBOC and RRSO, as well as healthcare infrastructure and culture, have changed since that systematic review was published in 2009 [7]. In Korea, the Health Insurance Review and Assessment Service approved *BRCA1/2* tests and RRSO for high-risk populations for HBOC in 2012 and 2013, respectively [14]. In addition, international interest in *BRCA* and preventive surgery has increased since the actress Angelina Jolie, who is a *BRCA1* carrier, received RRM in 2013 [13,14]. Despite prior research, there are gaps in explaining the process and factors associated with current RRSO decision-making.

This systematic review sought to identify the factors that influence the decision to undergo RRSO based on the existing literature. In addition, we explored the uptake rate of RRSO and the time interval between *BRCA* testing and RRSO among women at high risk for HBOC.

## Methods

Ethics statement: This study is a literature review of previously published studies and was therefore exempt from institutional review board approval.

This systematic review was conducted in accordance with the guidelines proposed by the Preferred Reporting Items of Systematic Review and Meta-Analysis (PRISMA) [15]. The study protocol was registered prospectively at the National Institute for Health Research (registration number: CRD42020188202).

### Literature search

To decide on the search terms, we reviewed 100 abstracts from relevant studies retrieved from MEDLINE. We combined keywords, such as “hereditary breast ovarian cancer,” “*BRCA*,” “risk-reducing surgery,” and “decision-making,” using “OR” for similar concepts and using “AND” for differing clusters. On July 6, 2020, the MEDLINE, Embase, CINAHL, and PsycINFO databases were searched, without any limitations on publication date (Supplementary Table 1).

### Study selection criteria

To identify suitable studies for this study purpose, PICO (population, intervention, comparison, outcome) criteria were defined and listed (Table 1). Based on PICO, the inclusion criteria were determined as follows: studies that (1) were about women at high risk for HBOC (e.g., *BRCA1/2* carriers, women with a family history of breast and/or ovarian cancers in multiple generations, and women who received genetic counseling for [risk of] breast and/or ovarian cancers); (2) reported on the factors influencing RRSO decision-making; and (3) were written in Korean or English. The exclusion criteria were as follows: (1) gray literature without peer review (e.g., conference abstracts, dissertations, and white reports); (2) animal experiments or preclinical experiments; (3) reviews, letters, and editorials; and (4) qualitative research.

Duplicate studies and gray literature were removed using a bibliography management program (EndNote X7, Clarivate, London, UK). Two authors (SYP and YLK) independently reviewed the titles and abstracts of identified studies, and selected studies according to the selection criteria.

### Risk-of-bias assessment

Two authors (SYP and YLK) independently assessed the quality of selected articles using the Risk of Bias for Nonrandomized Studies tool (RoBANS, version 2.0) [16]. RoBANS is a risk-of-bias tool for non-randomized trials (e.g., cohort studies, case-control, and before-and-after studies) that evaluates the selection of populations, confounding variables, measurement exposure, outcome blinding, incomplete data, and selective reporting. RoBANS has moderate reliability, acceptable validity, and is compatible with domains of the Cochrane risk-of-bias tool [16]. The results of evaluating these domains were presented as “low,” “high,” and “unclear” with Revman (version 5.0, Cochrane Community, Oxford, UK).

### Data analysis

One author (SYP) extracted the data from the selected literature using a predesigned form (first author and publication year, research country, population characteristics, study design and time of measurement, measurement, and significant or insignificant factors influencing RRSO), and another author (YLK) cross-checked the accuracy of data extraction. To show the overall significance of the reported factors, we synthesized data if the relevant factors were reported in two or more studies, and presented the ratio of the number of papers between significant and non-significant factors. The factors affecting decisions about RRSO were categorized into (1) demographic factors, (2) clinical factors, (3) family history of ovarian/breast cancer, (4) psychological factors, and (5) the objective risk of developing ovarian or breast cancer. To analyze the RRSO uptake rate, the intent and rate of RRSO were presented separately.

## Results

### Characteristics of the selected studies

**Figure 1.**
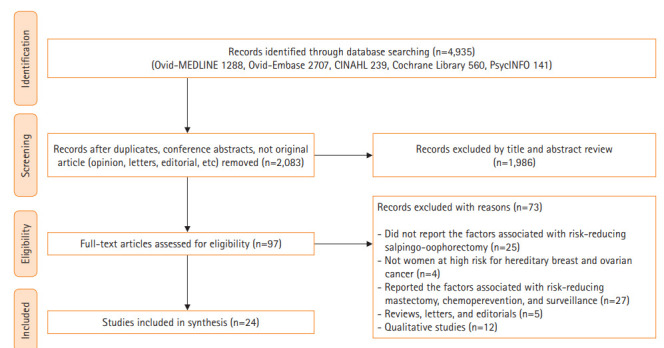
Flow diagram of study selection.

The chosen search strategies identified a total of 4,935 studies, from which 24 studies were finally included in the systematic review [11-14,17-36] (Table 2, Figure 1). The 24 studies included a total of 6,793 women (range, 42–1,241), and 10 were conducted in the United States, nine in Europe, two in Korea, and one each in Australia, Israel, and the United States/Canada/Europe together. Five studies (20.8%) were cross-sectional, 9 (37.5%) were prospective cohorts, and 10 (41.7%) used a retrospective cohort design. The selected literature were published between 1999 and 2019.

### Risk-of-bias assessment

**Figure 2.**
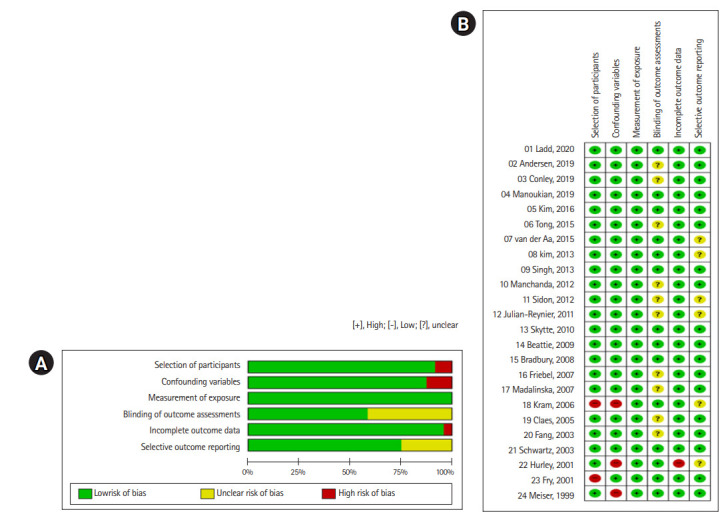
Risk-of-bias graph. (A) Risk of bias summary. (B) Risk of bias for selected studies

The risk of bias in the selected literature was moderately low (Figure 2). In particular, four domains were assessed as having a low risk of bias: selection of the population (91.7%), confounding variables (87.5%), measurement of exposure (100%), and incomplete data reporting (95.8%). For the blinding of the outcome evaluation, the risk of bias was unclear in 41.7% of articles, and for the section of selective reporting, 25% of articles were evaluated as unclear because they did not report whether the institutional review board had approved their research protocol or whether the researchers prospectively conducted their study after the protocol was registered. Although it was difficult to determine the level of bias of these two domains, we assumed that these two domains did not significantly affect the overall quality of the selected articles. Thus, all selected studies were included in the data analysis (Figure 2).

### Factors associated with RRSO decision-making

#### Demographic factors

Among the 19 studies that explored the effect of age on RRSO, 13 (68.4%) reported that older age was associated with a higher uptake of RRSO [11-13,21,23-26,28,30,31,33,35]. A Korean study [12] showed more instances of RRSO in women in their 40s than in their 50s, but the RRSO rate was higher in those over 50 years old in the United States [13] and France [25].

In 60.0% of the articles [11,23,26], more women chose RRSO if they had a child(ren). Marital status (80.0%) [20,29,32,33], employment status (100%) [12,14,20,32], education level (88.9%) [12-14,20,21,30,32,33], and race (80.0%) [13,20,21,32] were consistently non-significant factors for RRSO.

#### Clinical factors

All of the articles (100%) found that *BRCA1/2* gene mutations were a significant factor in decision-making about RRSO [5,19,23,30]; however, the type of *BRCA* mutation was not a significant factor in the articles (83.3%) that reported it [11,13,14,26,28]. A personal history of mastectomy was a significant factor in 80.0% of articles [13,18,22,27], while only 40.0% [12,23,27] and 33.3% of articles [14,23] reported that a personal history of breast cancer and menopausal status were significant factors for RRSO.

#### Family history of cancer

The vast majority of articles (81.8%) [12-14,20-22,28,32,36] reported that a family history of breast cancer influenced RRSO decision-making; however, a family history of ovarian cancer was not a significant factor in 66.6% of articles [12-14,19,20,22,33,36] (Tables 2, 3). Singh et al. [13] particularly suggested that although a family history of ovarian cancer was not a significant factor, the death of a mother or relative from pelvic or breast cancer affected RRSO decisions.

#### Psychological factors

The perceived risk/anxiety/concern for ovarian cancer was a significant factor in determining RRSO in most studies (91.7%, 11 of 12) that reported it [18-21,29,31-36]. Meanwhile, the perceived risk for breast cancer was not significant in two articles [21,32]. Four articles [20,29,33,34] consistently reported that positive perceptions of RRSO were related to the decision to undergo surgery. With regard to negative perceptions of RRSO, two studies [20,34] reported conflicting results. Cancer-related distress [20,21], anxiety [33,34], and depression [12,33] were not significant factors for RRSO in the two articles that reported those factors. The significance of health perceptions differed between the two studies [12,29].

#### Objective cancer risk

As a possible factor influencing RSSO decision-making, the reported objective cancer risk was the risk level evaluated by family cancer/genetic specialists based on a person’s family cancer history [34,36], and breast cancer risk assessment tool according to the person’s cancer status and family cancer history such as the BRCAPRO statistical model [20,21]. The objective cancer risk did not influence women’s RRSO decision-making in four articles [20,21,34,36].

### Rate and timing of RRSO decision-making

The RRSO rate was 11% to 87.2% across the 21 articles. In six studies [17,20,21,30,33,36], 11% to 61.6% of women at high risk for HBOC intended to undergo RRSO in the future (mean, 41.6%; 481 of 1,155 women). In 15 studies [11-14,18,22-29,31,32], 46.2% of women received RRSO (range, 23.4%–87.2%; 1,830 of 3,960 women) (Table 4).

Four articles [14,12,26,27] reported the length of time that elapsed between the *BRCA* test and RRSO (Table 4). Of the three articles that studied Koreans [12,14] and Americans [27], the mean time to decide was 2 to 7.3 months. Meanwhile, a Danish article [26] reported that it took 34 months to decide, and a Korean article [12] reported that the maximum time to decide was 64 months. In two Korean articles on *BRCA* carriers [12,14], the proportion of patients who received RRSO within 1 year after receiving a genetic consultation was reported to be high, at 85.7% [12] and 86.4% [14], respectively.

## Discussion

The goal of this study was to identify significant factors affecting RRSO decision-making among women at high risk of HBOC and to explore the uptake rate and decision timing for RRSO. Among the reviewed articles, 13 [11-13,21,23-26,28,30,31,33,35] suggested that older age was associated with the decision to undergo RRSO. Although six studies [17,19,20,29,32,36] did not find that age was significant, those studies analyzed age as a continuous variable; therefore, they failed to determine which age group received RRSO more. In this review, women in their 40s and 50s were more likely to undergo RRSO than other age groups in four studies [12,13,24,25], and the studies that analyzed age groups were more reliable than those that examined age as a continuous variable. Therefore, age is considered to have a significant influence on decision-making about RRSO.

Although our study and the previous systematic review [7] did not confirm whether having child(ren) [11,20,23,26,30] or menopause [12,14,23,29,33] affected RRSO decisions, childbirth and menopause status are important variables in the decision-making process for RRSO [7]. This is because women at high risk for HBOC fear surgical-related menopause [7], and fertility is important for women who want to become pregnant. Therefore, qualitative studies that explore how fertility and menopause affect decision-making through in-depth interviews would facilitate a deeper understanding of this issue.

In this review, a personal history of mastectomy was a significant factor affecting RRSO in 80.0% of the articles, but a history of breast cancer was not a significant factor in 40.0% of the studies. A previous systematic review also showed that women with breast cancer tended to select RRM more frequently than RRSO [7]. Further research is needed to examine whether breast cancer history is associated with RRM, and if having a mastectomy affects decision-making about RRSO.

Most of the selected studies showed that a family history of breast cancer (81.8%) and ovarian cancer (66.7%) were not associated with having RRSO. This result is supported by a systematic review reporting that RRM was more strongly affected than RSSO by family cancer history [7]. Therefore, a family history of ovarian/or breast cancer is assumed to be a more important factor in determining RRM than RRSO. In one article [13], RRSO was more likely to be chosen if a mother or relative had died from breast or ovarian cancer than simply having a family cancer history. Furthermore, Howard et al. [7] reported that RRM was more likely to be chosen based on experiences of first-degree relatives, especially mothers and sisters, rather than of having a family history of ovarian/breast cancer. Therefore, future studies should analyze the death of a close family member from cancer, as distinct from a family history of cancer.

Perceived risk of cancer is a well-known factor contributing to the choice to undergo risk-reducing surgery among women at high risk for HBOC [5,7,37]. Our study found that the perceived risk of ovarian cancer was the main motivation for choosing to undergo RRSO. However, the mechanism underlying cancer risk perception is still unknown [38]. Four articles [5,19,23,30] reported that *BRCA* carriers chose RRSO more frequently than non-carriers, which was an expected result. Although Padamsee et al. [5] suggested that the perception of RRSO could vary depending on the type of *BRCA* mutation, in this study there was no evidence that the type of *BRCA* mutation affected decision-making about RRSO [11,13,14,26,28]. Therefore, in-depth studies are needed to determine whether there are differences in the RRSO decision-making process depending on the *BRCA* mutation type [5].

A systematic review [7] found that psychological factors affected decisions about RRM, but we could not confirm whether psychological stability (e.g., cancer-related distress, anxiety, and depression) affected RRSO decision-making in this study. Therefore, further studies are needed to identify differences in psychological motivations for decisions about RRSO and RRM.

Previous qualitative studies showed that family factors were related to RRSO [7,39], and a systematic review found that spouses, family/friends, and doctors’ recommendations influenced the choice to undergo RRM [38]. However, we could not determine whether these factors were explored in quantitative studies related to RRSO. These gaps may suggest that family and interpersonal factors in RRSO decision-making have not been explored. However, family, friends, and communities influence the information obtained and the decision-making process. Therefore, further research is needed to identify the impact of these factors and to integrate the factors reported in qualitative studies.

In this review, objective cancer risk was not related to the decision to undergo RRSO. This result implies that women decide to undergo RRSO to reduce anxiety based on the perceived risk of ovarian cancer [40], rather than on objective information. In addition, the effect of genetic testing on RRSO decisions has not been reported to a sufficient extent. Therefore, it is necessary to confirm whether fully-informed decision-making is happening in the clinical setting.

The uptake rate of RRSO varied from 11% to 87.2% across the selected articles in this review. Among those who opted for RRSO, Koreans were younger than Europeans [12,13,25], and 71.2% to 87.2% of Danish [26] and Dutch [22, 29] women chose RRSO, which was a higher rate than that of women in other countries. This study also showed that Danish women took a longer time to make decisions than Koreans and Americans. These results imply that socio-cultural factors and national health care systems may affect RRSO decisions. This is supported by Padamsee et al. [5], who suggested that geographical differences, which may be a proxy for differences in health care infrastructure and cultural contexts, influence RRSO decisions. Therefore, further research is needed to examine how sociocultural factors and health care delivery systems affect RRSO decision-making and surgical timing.

The generalizability of the results of this systematic review is limited because we did not review the factors associated with RRSO from qualitative research. Nevertheless, this study is meaningful in that it provides fundamental information regarding factors affecting RRSO decisions based on current evidence. In particular, we found that the perceived risk of ovarian cancer, older age, and being a *BRCA* carrier are major factors affecting RRSO decision-making.

Based on the results of this study, we suggest the following: (1) considering that the decision process of RRSO is complex and involves various factors, it is necessary to identify how family factors, socio-cultural characteristics, and healthcare systems affect the decision process; (2) further studies are needed to confirm the significance of factors that have been reported in a few studies or have shown contradictory results across articles; and (3) interventions should be developed based on information about objective cancer risk.

## Figures and Tables

**Table 1.** PICO framework

Population (P)	Women at high risk for HBOC include the following:
	- *BRCA1/2* carriers
	- Women with a family history of breast and/or ovarian cancers in multiple generations
Intervention (I)	Studies that analyzed factors associated with RRSO decision-making
Comparators (C)	Factors associated with decision-making of the following:
	- Chemoprevention
	- Surveillance
	- Risk-reducing mastectomy
Outcomes (O)	Factors associated with RRSO decision-making among women at high risk for HBOC
	Rates of RRSO decision-making
	Timing of RRSO decision-making

*BRCA*: Breast cancer susceptibility gene; HBOC: hereditary breast and ovarian cancer; RRSO: risk‐reducing salpingo‐oophorectomy.

**Table 2.** Main characteristics of selected studies (N=24)

First author (year)	Country (period of data collection)	Population characteristics	Study design & time of measurement (or follow-up)	Measurement	Significant factors for RRSO	Non-significant factors for RRSO
Ladd (2020) [20]	USA (NR)	· 168 Women who received genetic counseling for HBOC (newly diagnosed breast cancer, probands or relatives with a *BRCA* mutation)	· Cross-sectional study (self-report)	I: intention for RRSO	· Perceived advantages of RRSO	· Age
			· Post-counseling	C: no intention for RRSO	· Lower score of perceived disadvantages of RRSO	· Having children
					· Perceived risk for ovarian cancer	· Race
					· Lower ambiguity aversion	· Education
					· HBOC relatives had a higher intention for RRSO than probands of HBOC	· Marital status
						· Employment status
						· Insurance status
						· Family history of breast cancer or ovarian cancer
						· Uncertainty
						· Cancer distress
						· Objective cancer risk
						· Cancer stage at diagnosis
Andersen (2019) [19]	USA (2010–2013)	· 1,100 Women at high risk for ovarian cancer (234 *BRCA1/2* mutation carriers and 866 high-risk pedigrees)	· Prospective cohort study (self-report)	I: RRSO	· *BRCA1/2 *carrier status	· Age
			· Up to 72 months	C: no RRSO	· Worry about ovarian cancer	· Family history of ovarian cancer
					· Experienced false positive screening events for high-risk pedigree	
Conley (2019) [17]	USA (2016–2017)	· 103 Women at high risk for breast cancer	· Cross-sectional study (self-report)	I: intention for RRSO	· *BRCA1/2* carrier status	· Age
			· NA	C: no intention for RRSO		
Manoukian (2019) [18]	Italy (2008–2015)	· 128 *BRCA1/2* mutation carriers who received pre-/post-test genetic counseling	· Prospective cohort study (self-report)	I: RRSO	· Having a child	NR
			· 15 months after the post-test genetic counseling	C: surveillance	· Mastectomy history for cancer therapy	
					· Worry about ovarian cancer	
					· A higher number of family members with cancer	
					· Lower general health perception	
					· Greater feeling of being full of energy	
Kim (2016) [14]	South Korea (2007–2014)	· 42 *BRCA1/2* mutation carriers	· Retrospective cohort study (medical records review)	I: RRSO	· Menopausal at the time of genetic counseling	· Age
			· Up to 36 months	C: surveillance	· Consultation with gynecologic oncologists	· Body mass index
						· Having child
						· Education
						· Employment status
						· Comorbidities
						· The type of *BRCA* mutation (*BRCA1* and *BRCA2*)
						· Personal history of breast cancer
						· Family history of breast cancer
						· Family history of ovarian cancer
Tong (2015) [21]	USA (2005–2012)	· 614 Women who received genetic counseling for HBOC	· Cross-sectional study (self-report)	I: intention for RRSO	· Age: older	· Race
			· Pre-counseling	C: no intention for RRSO	· Family history of ovarian cancer	· Education
					· Perceived risk for ovarian cancer	· Cancer distress
					· Perceived risk of a *BRCA1/2* mutation	· Perceived risk for breast cancer
						· Personal history of breast or ovarian cancer
						· Family history of cancer
						· Objective cancer risk
						· Knowledge of cancer genetics
						· Hormone receptor positivity
						· Decisional conflict
van der Aa (2015) [22]	The Netherlands (2011–2013)	· 218 Women who had familial history or *BRCA1/2* mutation	· Retrospective cohort study (medical records review)	I: RRSO	· History of preventive mastectomy	· Personal history of breast cancer
			· NR	C: no RRSO		· Personal history of any cancer
						· Family history of breast cancer
						· Family history of ovarian cancer
Kim (2013) [12]	South Korea (2003–2011)	· 71 *BRCA1/2* mutation carriers eligible for RRSO	· Retrospective cohort study (medical records review)	I: RRSO	· Age: more women in their 40s than in their 50s	· Menopausal status
			· Up to 64 months	C: no RRSO	· Personal history of breast cancer	· Body mass index
						· Family history of breast cancer
						· Family history of ovarian cancer
						· Health perception
						· Education
						· Employment status
						· Economic status
						· Depression status
Singh (2013) [13]	USA (1998–2010)	· 136 Unaffected women with *BRCA* mutations	· Retrospective cohort study (medical records review)	I: RRSO	· Age at time of surgery ≥ 50 years	· Age at the time of genetic testing
			· Up to 132 months	C: surveillance	· Having children	· Race
					· Mastectomy	· Education
					· History of relative dying from breast cancer	· Personal history of breast biopsy
					· History of a mother lost to pelvic cancer	· The type of *BRCA* mutation (*BRCA1* and *BRCA2*)
					· Genetic testing ≥ 2005	· Family history of cancer: first-degree relatives with breast cancer
						· Family history of cancer: first-degree relatives with ovarian cancer
						· Previous cosmetic surgery
Manchanda (2012) [23]	UK (2004–2009)	· 1,241 Women who received genetic counseling for HBOC (287 *BRCA1/2* mutation carriers and 866 with high-risk pedigrees)	· Prospective cohort study (medical records review)	I: cumulative incidence function of RRSO	· Age: older	NR
			· Up to 80 months	C: surveillance	· Having children	
					· *BRCA1/2* carrier status	
					· Menopausal status: postmenopausal	
					· Personal history of breast cancer	
					· Family history of breast cancer	
Sidon (2012) [24]	UK (1996–2011)	· 700 *BRCA1/2* mutation carriers	· Prospective cohort study (medical records review)	I: RRSO	· Age: 40–59 years than over the 60s	NR
			· 60 months post-*BRCA* testing	C: no RRSO	· The type of *BRCA* mutation (more *BRCA1* mutation carriers had RRSO than *BRCA2*)	
Julian-Reynier (2011) [25]	France (2000–2006)	· 101 *BRCA1/2* mutation carriers	· Prospective cohort study (self-report)	I: RRSO	· Age: over 50 years old (30s: 18.4%, 40s: 55.6%, 50s: 76.2%)	NR
			· Up to 60 months	C: no RRSO		
Skytte (2010) [26]	Denmark (1998–2008)	· 306 Women (*BRCA1/2* mutation carriers) with no personal history of ovarian or breast cancer	· Retrospective cohort study (medical records review)	I: RRSO	· Age: 35–60 years than <35 years	· The type of *BRCA* mutation (*BRCA1* and *BRCA2*)
			· 6–120 months post-*BRCA* testing	C: no RRSO	· Having children	
Beattie (2009) [27]	USA (1996–2006)	· 240 Women (*BRCA1/2* mutation carriers)	· Retrospective cohort study (medical records review)	I: RRSO	· Age was positively associated with surgery uptake until age 60 years, with women 50–59 years old most likely to undergo RRSO	NR
			· 44.4 months (median)	C: no RRSO	· Personal history of breast cancer	
					· Mastectomy history for prevention	
Bradbury (2008) [11]	USA (1996–2003)	· 88 Women (*BRCA1/2* mutation carriers)	· Retrospective cohort study (medical records review)	I: RRSO	· Age: older than 40 years	· The type of *BRCA* mutation (*BRCA1* and *BRCA2*)
			· Up to 84 months	C: no RRSO	· Race: white	· Personal history of breast cancer
					· Having children	· Mastectomy
					· Family history of ovarian cancer	
					· Family history of breast cancer: within one or two relatives	
Friebel (2007) [28]	USA, Canada, and Europe (1994–2006)	· 537 Women (*BRCA1/2* mutation carriers)	· Prospective cohort study (self-report, medical records review)	I: RRSO	· Age ≥ 40 years compared with aged < 40 years (68% vs. 43%)	· The type of *BRCA* mutation (*BRCA1 *and *BRCA2*)
			· More than 6 months post-disclosure of *BRCA* testing	C: no RRSO	· Having a child for *BRCA1* carriers	· Family history of breast cancer
					· Family history of ovarian cancer for *BRCA2* carriers	
Madalinska (2007) [29]	The Netherlands (2002–2004)	·160 Women (*BRCA1/2 *mutation carriers)	· Prospective cohort study (self-report)	I: RRSO	· Education: under high school reported higher RRSO than advanced vocational/university	· Age
			· Up to 12 months	C: surveillance	· Perceived incurability of ovarian cancer	· Marital status
					· Perceived benefits of RRSO	· Menopausal status
					· Lower general health perceptions	· Worry about ovarian cancer
						· Perceived risk for ovarian cancer
Kram (2006) [30]	Israel (1995–2001)	· 99 Women who had *BRCA* tests	· Retrospective cohort study (mail, self-report)	I: intention for RRSO	· *BRCA1/2* carrier status	· Personal history of breast cancer
			· 12–48 months after receiving *BRCA* test	C: no intention for RRSO	· Age: older than 50 years	· Education
						· Having children
Claes (2005) [31]	Belgium (1999–2003)	· 68 Women who received genetic counseling for HBOC (34 *BRCA1/2* mutation carriers and 34 non-carriers)	· Prospective cohort study (self-report)	I: RRSO	· Age: older	
			· 12 months after receiving *BRCA* test	C: no RRSO	· Perceived risk for ovarian cancer	
Schwartz (2003) [32]	USA	· 289 High-risk women who underwent genetic counseling and testing for *BRCA1/2*	· Prospective cohort study (self-report)	I: RRSO	· Family history of cancer: first-degree relatives with ovarian cancer	· Age (standard: 35 years)
	(1995–2000)		· 12 months after receiving *BRCA* test	C: surveillance	· Perceived risk for ovarian cancer	· Education
						· Race
						· Marital status
						· Employment status
						· Menopausal status
						· Personal history of breast cancer
						· Family history of cancer: first-degree relatives with breast cancer
						· Perceived risk for breast cancer
Fang (2003) [33]	USA (NR)	· 76 Women enrolled in a familial cancer risk assessment program	· Cross-sectional study (self-report)	I: intention for RRSO in 12 months	· Age: older	· Education
			· Following familial cancer education and genetic counseling	C: no intention for RRSO	· Perceived risk of ovarian cancer	· Marital status
					· Perceived benefits of RRSO	· Family history of ovarian cancer
						· Anxiety
						· Depression status
Hurley (2001) [34]	USA (1997)	· 94 Women who received genetic counseling for family history of ovarian cancer	· Retrospective cohort study (telephone interview, self-report)	I: interest in RRSO in the future	· Perceived benefits of RRSO	· Anxiety of cancer
			· 12 months after genetic counseling	C: no interest to RRSO in the future	· Reducing anxiety/uncertainty	· Perceived efficacy of RRSO
					· Stress-related ideation	· Risks of RRSO
						· Objective cancer risk
Fry (2001) [35]	UK (1999)	·58 Women (30 had RRSO, 28 attended a screening program for HBOC)	· Retrospective cohort study (self-report)	I: important factors for RRSO	· Age: older	· Other family members choosing prophylactic surgery
			· 12–60 months		· Reducing cancer worry	· Partner’s attitude
					· Reducing risk of ovarian cancer	· Desire to have children
					· Worry about effectiveness of ovarian screening	· Fear of menopausal symptoms
					· The importance of recovery time	· Need to feel like a woman
					· The importance of loss of periods	· Dislike of ovarian screening methods
Meiser (1999) [36]	Australia (1996–1999)	· 95 High-risk women who attended familial cancer clinics	· Retrospective cohort study (mail, self-report)	I: interest in RRSO	· Anxiety of breast/ovarian cancer	· Age
			· Prior to attendance at a familial cancer clinic			· Number of first- and second-degree relatives with breast or ovarian cancer
						· Objective cancer risk

*BRCA*: Breast cancer susceptibility gene; C: comparator; HBOC: hereditary breast and ovarian cancer; I: intervention; NA: not available; NR: not reported; RRSO: risk‐reducing salpingo‐oophorectomy.

**Table 3.** Factors associated with the decision to undergo RRSO among women at high risk for hereditary breast and ovarian cancer

Factor		Significant factors for RRSO		Non-significant factors for RRSO	
		No. of articles^†^	References	No. of articles^†^	References
Demographic factors	· Age	13/19	[11-13,21,23-26,28,30,31,33,35]	6/19	[17,19,20,29,32,36]
	· Marital status	1/5	[11]	4/5	[20,29,32,33]
	· Having child(ren)	3/5	[11,23,26]	2/5	[20,30]
	· Employment status	0/4	Not reported	4/4	[12,14,20,32]
	· Education level	1/9	[29]	8/9	[12-14,20,21,30,32,33]
	· Race	1/5	[11]	4/5	[13,20,21,32]
Clinical factors	· *BRCA1/2* carrier status	4/4	[17,19,23,30]	0/4	Not reported
	· The type of *BRCA* (*BRCA1* vs. *BRCA2*)	1/6	[24]	5/6	[11,13,14,26,28]
	· Menopausal status	2/5	[14,23]	3/5	[12,29,32]
	· Mastectomy	4/5	[13,18,22,27]	1/5	[11]
	· Personal history of breast cancer	3/9	[12,23,27]	6/9	[11,12,21,22,30,32]
Family history of cancer	· Family history of breast cancer	2/11	[11,23]	9/11	[12-14,20-22,28,32,36]
	· Family history of ovarian cancer	4/12	[11,21,28,32]	8/12	[12-14,19,20,22,33,36]
Psychological factors	· Perceived risk/worry/anxiety for ovarian cancer	11/12	[18-21,29,31-36]	1/12	[29]
	· Perceived risk for breast cancer	0/2	Not reported	2/2	[21,32]
	· Perceived advantages of RRSO	4/4	[20,29,33,34]	0/4	Not reported
	· Perceived disadvantages of RRSO	1/2	[20]	1/2	[34]
	· General health perceptions	1/2	[29]	1/2	[12]
	· Cancer distress	0/2	Not reported	2/2	[20,21]
	· Anxiety	0/2	Not reported	2/2	[32,34]
	· Depression	0/2	Not reported	2/2	[12,33]
Objective cancer risk		0/4		4/4	[20,21,34,36]

*BRCA*: Breast cancer susceptibility gene; RRSO: risk-reducing salpingo-oophorectomy.

† Reported in articles/total articles.

**Table 4.** RRSO rate and timing among women at high risk for HBOC

Category / First author (year)	Country	Follow-up (month)	RRSO, n (%)	Timing for RRSO (month), mean (range)
Intention for RRSO in women at high risk for HBOC				
Ladd (2020) [20]	USA	NR	103/168 (61.3)	NR
Conley (2019) [17]	USA	NR	11/103 (10.7)	NR
Tong (2015) [21]	USA	NR	261/614 (42.5)	NR
Kram (2006) [30]	Israel	12–48	61/99 (61.6)	NR
Fang (2003) [33]	USA	NR	26/76 (34.2)	NR
Meiser (1999) [36]	Australia	NR	19/95 (20.0)	NR
Total			481/1,155 (41.6)	
RRSO in women at high risk for HBOC				
Manoukian (2019) [18]	Italy	15	55/128 (43.0)	NR
Kim (2016) [14]	South Korea	36	22/42 (52.4)	7.3 (0.6–33.9)
van der Aa, 2015 [22]	The Netherlands	NR	190/218 (87.2)	NR
Kim (2013) [12]	South Korea	64	21/71 (29.6)	2 (0–64)
Singh (2013) [13]	USA	NR	71/136 (52.2)	NR
Manchanda (2012) [23]	UK	80	265/1,133 (23.4)	NR
Sidon (2012) [24]	UK	60	309/700 (44.1)	NR
Julian-Reynier (2011) [25]	France	60	43/101 (42.6)	NR
Skytte (2010) [26]	Denmark	6–120	218/306 (71.2)	34
Beattie (2009) [27]	USA	6–120	122/240 (50.8)	4
Bradbury (2008) [11]	USA	84	62/88 (70.5)	NR
Friebel (2007) [28]	North America, EU	≥6	297/537 (55.3)	NR
Madalinska (2007) [29]	Netherlands	12	118/160 (73.8)	NR
Claes (2005) [31]	Belgium	12	16/21 (75.0)	NR
Schwartz (2003) [32]	USA	12	21/79 (26.6)	NR
Total			1,830/3,960 (46.2)	

HBOC: Hereditary breast and ovarian cancer; NR: not reported; RRSO: risk-reducing salpingo-oophorectomy.

## Supplementary materials

Further details on supplementary materials are presented online (available at: https://doi.org/10.4069/kjwhn.2020.11.19).

Supplementary Table 1. Search strategy.
