# Supplementary material for: Factors associated with the decision to undergo risk-reducing salpingo-oophorectomy among women at high risk for hereditary breast and ovarian cancer: a systematic review
Source: Korean J Women Health Nurs. 2020 Dec 9;26(4):285–99. doi: 10.4069/kjwhn.2020.11.19 (PMC9328615; doi:10.4069/kjwhn.2020.11.19)
Supplement: Supplementary Table 1. — Search strategy. [file kjwhn-2020-11-19-suppl.pdf]

Supplementary Table 1. Search strategy

Databases	No.	Search term	Results
Ovid-MEDLINE	1	((hereditary OR genetic$ OR predispose$) AND (breast cancer OR ovarian cancer)).ti,ab,hw. OR BRCA. ti,ab,hw. OR Genes, BRCA1/ OR Genes, BRCA2/ OR BRCA2 Protein/	41,264
	2	((prevent$ OR risk reducing OR prophylact$) AND (chemoprevention OR surveillance OR surgery OR surgical OR surgeries OR screening OR operation OR opportunit$ OR mastectomy OR oophorectomy OR salpingectomy OR hormone)).ti,ab,hw.	292,716
	3	(consider$ OR decision OR decid$ OR perspective OR perceive$ OR determin$ OR prefer$ OR reason).ti,ab,hw.	6,141,512
	4	1 AND 2 AND 3	1,313
	5	4 NOT (animal/ NOT (animal/AND human/))	1,303
	6	5 NOT (letter/ OR editorial/ OR comment/)	1,288
	Total		1,288
Ovid-EMBASE	1	((hereditary OR genetic$ OR predispose$) AND (breast cancer OR ovarian cancer)).ti,ab,hw. OR BRCA. ti,ab,hw.	80,896
	2	((prevent$ OR risk reducing OR prophylact$) AND (chemoprevention OR surveillance OR surgery OR surgical OR surgeries OR screening OR operation OR opportunit$ OR mastectomy OR oophorectomy OR salpingectomy OR hormone)).ti,ab,hw.	473,365
	3	(consider$ OR decision OR decid$ OR perspective OR perceive$ OR determin$ OR prefer$ OR reason).ti,ab,hw.	8,085,546
	4	1 AND 2 AND 3	2,732
	5	4 NOT (animal/ NOT (animal/ AND human/))	2,731
	6	5 NOT (letter/ OR editorial/ OR comment/)	2,707
	Total		2,707
CINAHL	1	(AB ((BRCA OR ((hereditary OR genetic) AND (breast OR ovarian) AND (cancer)))) OR TI ((BRCA OR ((hereditary OR genetic) AND (breast OR ovarian) AND (cancer))))) AND (AB ((((Preventive OR risk reducing OR prophylactic) AND (surgery OR surgical OR surgeries OR screening OR operation OR opportunity OR oophorectomy OR salpingectomy OR hormone)) OR chemoprevention OR surveillance))) OR TI ((((Preventive OR risk reducing OR prophylactic) AND (surgery OR surgical OR surgeries OR screening OR operation OR opportunity OR oophorectomy OR salpingectomy OR hormone)) OR chemoprevention OR surveillance)))) AND (AB ((decision OR decide OR perspective OR perceived OR determine OR determinant OR prefer OR reason))) OR TI ((decision OR decide OR perspective OR perceived OR determine OR determinant OR prefer OR reason))))	239
	Total		2397
Cochrane library	1	(BRCA OR ((hereditary OR genetic) AND (breast OR ovarian) AND (cancer))) AND (preventive OR prevention OR risk reducing OR prophylactic OR hormone OR chemoprevention OR surveillance OR surgery OR surgical OR surgeries OR screening OR operation OR opportunity OR mastectomy OR oophorectomy OR salpingectomy OR hormone) AND (consider OR decision OR decide OR perspective OR perceived OR determine OR determinant OR prefer OR reason)	560
	Total		560
PsychINFO	1	((hereditary OR genetic$ OR predispose$) AND (breast cancer OR ovarian cancer)).ti,ab,hw,mh. OR BRCA. ti,ab,hw,mh.	1,108
	2	((prevent$ OR risk reducing OR prophylact$) AND (chemoprevention OR surveillance OR surgery OR surgical OR surgeries OR screening OR operation OR opportunit$ OR mastectomy OR oophorectomy OR salpingectomy OR hormone)).ti,ab,hw,mh.	25,025
	3	(consider$ OR decision OR decid$ OR perspective OR perceive$ OR determin$ OR prefer$ OR reason). ti,ab,hw,mh.	1,475,064
	4	1 AND 2 AND 3	141
	Total		141

https://doi.org/10.4069/kjwhn.2020.11.19
